# Determination of the Minimum Inhibitory Concentrations of Alexidine and Chlorhexidine Against Enterococcus faecalis and Candida albicans: An In Vitro Study

**DOI:** 10.7759/cureus.2221

**Published:** 2018-02-23

**Authors:** Fatma Kermeoglu, Umut Aksoy, Atakan Kalender, Meltem D Oztan, Ece I Oguz, Mehmet Kıyan

**Affiliations:** 1 Dentistry, Near East University; 2 Endodontics, Near East University; 3 Endodontics, Ankara university; 4 Prosthodontics, Ankara university; 5 Medical Microbiology, Ankara university

**Keywords:** alexidine, chlorhexidine, c. albicans, endodontic irrigants, e. faecalis

## Abstract

Introduction: The root canal system must be mechanically instrumented and chemically cleaned using various antimicrobial irrigants in a sequential manner or in combination for the elimination of necrotic pulp tissue and reducing the number of root canal bacteria. For this reason, new methods and materials are continuously being developed to achieve the objectives of endodontic treatment.

Materials and Methods: E. faecalis (ATCC 29212) and C. albicans (ATCC 90028) standard strains were used for this study. Colonies of E. faecalis and C. albicans were harvested from the agar plates and suspended in 4 mL of phosphate buffered saline (PBS). Microorganisms were diluted to obtain a suspension of approximately 108 colony-forming units/mL (CFU/mL) in sterile PBS using McFarland standard tubes no. 0.5.

Results: After a two-minute contact time, all alexidine (ALX) concentrations used in this study eradicated all E. faecalis strains, while chlorhexidine (CHX) didn’t kill 100% of E. faecalis at 0.25% and lower concentrations even after a five-minute contact time. ALX also eradicated C. albicans at all concentrations even after a one-minute contact time. CHX showed antifungal activity against C. albicans at all concentrations higher than 0.031% after a one-minute contact time.

Conclusion: A 0.0156% concentration of ALX can be a good alternative to CHX as an irrigation solution in endodontic treatment when used for one minute against E. faecalis and C. albicans.

## Introduction

Microorganisms and their by-products are regarded as the major cause of the formation and development of pulpal and periapical alterations [1-2]. Bacteria in the root canal organize either as free-floating single cells or organize communities attached to each other or to the areas of inaccessible root canal walls to form a biofilm [2-3]. Enterococcus faecalis (E. faecalis), a gram-positive facultative anaerobic microorganism capable of invading the dentinal tubules, is more likely to be found in persistent infections than in primary infections. Hence, many studies have been carried out onE. faecalis-infected dentin blocks to determine the antimicrobial activity of intracanal irrigants [4-6]. Another important consideration in endodontic treatment is the elimination of fungi from the root canal system. The largest proportion of the fungal oral microbiota is made up of the Candida species. Although studies demonstrated that fungi are not common members of the microbiota in primary endodontic infections, Candida albicans (C. albicans) has been associated with root canal infections resistant to nonsurgical therapy and potent pathogens to infect periapical lesions as E. faecalis [7].

The primary objective of endodontic treatment is to clean, disinfect, and seal the root canal system from sources of infection [3]. Therefore, to eliminate necrotic pulp tissue and reduce the number of root canal bacteria, the root canal system must be mechanically instrumented and chemically cleaned using various antimicrobial irrigants in a sequential manner or in combination [8- 9]. Although chemomechanical preparation does not result in the total elimination of bacteria from infected root canals, it is the most important step in endodontic disinfection. For this reason, new methods and materials are continuously being developed to achieve the objectives of endodontic treatment [10].

The use of irrigants helps provide residual antimicrobial activity, avoiding the negative impact that a bacteria invasion would have on the success of an endodontic procedure [11]. Sodium hypochlorite (NaOCl) is the most frequently used root canal irrigation solution, which has a wide-ranging activity against endodontic microorganisms and dissolves necrotic tissues and debris through a complex biochemical process [3,7,12]. Despite the favorable qualities of NaOCl, it has significant disadvantages, such as bad odor and taste, cytotoxicity, does not impart antimicrobial substantivity, and forms para-chloroaniline (PCA) after interaction with chlorhexidine (CHX) [13-15].

CHX is a cationic bisbiguanide disinfectant, which has also been widely used as an endodontic final irrigation solution, as it has antimicrobial action, proven substantivity, and inhibits the adherence of certain bacteria to dentin [16-18]. It has broad antimicrobial spectrum (i.e. against gram-positive/negative bacteria and fungi) at a concentration of 2% [19-20].

Alexidine (ALX), similar to CHX, is also a bisbiguanide, which has been used as an antiseptic in mouthwashes and as a disinfectant in contact lens solutions. It has an affinity for major virulence factors, such as bacterial lipopolysaccharide (LPS) and lipoteichoic acid (LTA) [6]. It differs chemically from CHX by the presence of two hydrophobic ethyl-hexyl end groups, as opposed to the p-chlorophenyl moieties of CHX [21-22]. Its structure provides hydrophobic penetration into membrane lipids and electrostatic adhesion to the negative sites of cell membranes [23-24]. In comparison with CHX, it has faster bactericidal activity and bacterial permeabilization [22,25]. Additionally, the interaction of ALX and NaOCl does not form an insoluble PCA precipitate [6].

The purpose of this study was to determine the minimum inhibitory concentration (MIC) values of CHX and ALX against *E. faecalis* and *C. albicans* for the effective use of ALX as a good alternative to CHX for root canal irrigation sequentially or in combination with NaOCl.

## Materials and methods

Microorganisms

The E. faecalis (ATCC 29212) and C. albicans (ATCC 90028) standard strains were used for this study. For the regeneration of the lyophilized strains, 0.1 mL of the phosphate buffered saline (PBS) diluted E. faecalis (ATCC 29212) and C. albicans (ATCC 90028) were cultivated in 2 mL of brain-heart infusion broth (BHIB, HiMedia Lab., Pvt., Ltd. Mumbai, India). E. faecaliswas subcultured on 5% blood agar (Lab M, Lancashire, United Kingdom) and C. albicans was subcultured on Sabouraud dextrose agar (SDA, Lab M, Lancashire, United Kingdom) plates and incubated at 37 °C for 24 hours. Microbial strains were confirmed by Gram’s stain and by colonial and growth characteristics.

Preparation of bacterial suspensions

Colonies of E. faecalisand C. albicanswere harvested from the agar plates and suspended in 4 mL of PBS. Microorganisms were diluted to obtain a suspension of approximately 108 colony-forming units/mL (CFU/mL) in sterile PBS using McFarland standard tubes no 0.5. The number of microorganisms per milliliter was determined by the optical densities of cultures at a wavelength of 600 nmol L-1 (OD600) by spectrophotometer (IMPLEN, Munich, Germany).

Microbial suspensions in PBS were vortexed for one minute. After 10-fold serial dilutions in PBS were prepared, 0.1 mL aliquots were plated onto blood agar plates at 37 °C for 24 hours. Colonies were counted, and a log transformation was calculated.

Root canal irrigants

All CHX concentrations were prepared by diluting a 20% solution of CHX (Sigma-Aldrich, St Louis, MO, USA) with sterile distilled water. An initial solution of 40 mg ALX dihydrochloride powder (Santa Cruz Biotechnology, Inc, Dallas, Texas, USA) was dissolved in 10 ml of 60% ethanol and 4% ALX was prepared. Nine two-fold dilutions were made from this solution in sterile distilled water to obtain a concentration of 0.0039% ALX in a microtiter plate. The activity of ethanol on the biofilms at the concentrations used in the ALX solutions was formerly determined, showing that ethanol achieved a reduction of ≤0.5 logarithmic units at all the concentrations tested, which is considered inconsequential. All the dilutions were carried out using sterile distilled water and were stored at room temperature until use, for no more than 60 minutes. The contact times of the irrigants on biofilms were one, two, three, and five minutes.

Susceptibility testing of E. faecalis and C. albicans

The susceptibility tests were done on a microtiter plate known as the “challenge plate.” The dilutions of the irrigating solutions were placed along the length of the plate, allowing the first and last wells of each row to serve as the sterility and growth controls, respectively. The peg lid was submerged in the challenge plate (for one, two, three, and five minutes.). After exposure, 100 µl E. faecalis or C. albicans suspensions were placed in a microtiter recovery plate, diluted serially in 0.9% saline and 50 µl aliquots were plated on blood agar or SDA plates for viable cell counting. Ten replicates per irrigant concentration, period, and bacteria were performed. After a 24-hour incubation at 37 °C, subcultures were performed on blood agar or SDA plates from the wells, showing no visible growth in order to determine the bactericidal concentration of the irrigant.

The term “eradication” was used to denote the death of 100% of the bacterial population.

## Results

The results of the antimicrobial activity of ALX and CHX against E. faecalis and C. albicans are shown in Table 1 and Table 2, respectively. ALX eradicated E. faecalis at all concentrations higher than 0.0078% after a one-minute contact time. CHX showed antimicrobial activity against E. faecalis at all concentrations higher than 0.5% after a one-minute contact time. After a two-minute contact time, all ALX concentrations used in this study eradicated all E. faecalis strains, while CHX didn’t kill 100% of E. faecalisat 0.25% and lower concentrations even after a five-minute contact time. ALX also eradicatedC. albican*s *at all concentrations higher than 0.0156% only after a one-minute contact time. While CHX showed antifungal activity against C. albicans at all concentrations higher than 0.031% after the one-minute contact time, the 0.0039% concentration of CHX did not eradicate C. albicans even after the five-minute contact time.

**Table 1:** Antimicrobial activity of ALX and CHX against E. faecalis Antimicrobial activity of ALX and CHX against E. faecalisafter different contact times: mean (SD) ALX: alexidine; CHX: chlorhexidine

	Concentrations %	E. faecalis (ATCC 29212)			
		CFU/ml (±SD)			
		One min.	Two mins.	Three mins.	Five mins.
Alexidine	0.125	E	E	E	E
	0.0625	E	E	E	E
	0.031	E	E	E	E
	0.0156	E	E	E	E
	0.0078	0.20x10 (±0.08x10)	E	E	E
	0.0039	0.55x10^2 ^(±0.20x10^2^)	E	E	E
Chlorhexidine	4	E	E	E	E
	3	E	E	E	E
	2	E	E	E	E
	1	E	E	E	E
	0.5	0.12x10 (±0.12x10)	E	E	E
	0.25	2.55x10^3 ^(±2.56x10^3^)	1.13x10^2 ^(±1.12x10^2^)	0.22x10 (±0.09x10)	0.02x10 (±0.05x10)
	0.125	1.01x10^4^ (±0.57x10^4^)	3.33x10^3 ^(±3.28x10^3^)	3.35x10 (±1.23x10)	0.42x10 (±0.22x10)
	E: Eradication				

**Table 2:** Antimicrobial activity of ALX and CHX against C. albicans Antimicrobial activity of ALX and CHX against C. albicans after different contact times: mean (SD) ALX: alexidine; CHX: chlorhexidine

	Concentrations %	C. albicans (ATCC 90128)			
		CFU/ml (±SD)			
		One min.	Two mins.	Three mins.	Five mins.
Alexidine	0.125	E	E	E	E
	0.0625	E	E	E	E
	0.031	E	E	E	E
	0.0156	E	E	E	E
	0.0078	E	E	E	E
	0.0039	E	E	E	E
Chlorhexidine	0.25	E	E	E	E
	0.125	E	E	E	E
	0.0625	E	E	E	E
	0.031	1.67x10 (±1.88x10)	0.05x10 (±0.1x10)	E	E
	0.0156	4.25x10^2 ^(±2.32x10^2^)	2.30x10 (±1.66x10)	E	E
	0.0078	4.62x10^3 ^(±2.49x10^3^)	1.40x10^2^ (±1.24x10^2^)	0.50x10 (±0.46x10)	E
	0.0039	3.70x10^5 ^(±1.44x10^5^)	7.47x10^3 ^(±1.14x10^3^)	6.60x10^2^ (±2.01x10^2^)	0.25x10 (±0.19x10)
	E: Eradication				

## Discussion

This study aims to evaluate the antimicrobial activity of two cationic molecules, ALX and CHX, against E. faecalis and C. albicans, which are the commonly isolated microorganisms from infected root canals.

The introduction of new antibacterial agents increases the need for antimicrobial sensitivity tests, which has received criticism, as all may show variability, particularly if insufficient attention is given to the careful standardization of technique. The tube dilution method appears to be one of the most reliable methods for determining the levels of microbial resistance to an antimicrobial agent [26]. The minimal/minimum inhibitory concentration (MIC) is the lowest concentration of an antimicrobial agent that inhibits the visible growth of bacteria. The lowest concentration preventing bacterial growth is considered the MIC [27].

We found that both ALX and CHX solutions were effective at low concentrations against E. faecalis and C. albicans. However, the MIC values for ALX were lower than CHX. In this study, after the two-minute contact time, all ALX dilutions eradicated E. faecalis, whereas only 0.5% and higher concentrations of CHX eradicated E. faecalis. The results of the present study are in accordance with the findings of Barrios et al. who showed that 2% and 1% ALX used for one minute provide longer antimicrobial substantivity against E. faecalis than CHX when applied to 2% and 0.5% [25]. However, Kim et al. found that 1% ALX was effective against E. faecalis infection and there was no difference in antibacterial activity against E. faecalis between 1% ALX and 2% CHX [6].

In the present study, ALX showed antifungal activity against C. albicans at all concentrations only after a one-minute contact time, whereas only 0.031% and higher concentrations of CHX showed antifungal activity after a one-minute contact time. Our results also indicated that the antifungal efficacy of ALX was significantly better than CHX againstC. albicans. These findings are in agreement with Roberts & Addy, who compared the bactericidal properties of 0.2% CHX gluconate mouthwash and 0.035% ALX mouthwash [27]. They concluded that both antiseptics were similar in action, being effective against C. albicans at low concentrations. The results of the present study are also in line with the findings of Yanai et al. regarding the antifungal efficacy of ALX [28]. They found that ALX manifests robust and rapid antimicrobial activity against bacteria and fungi under ideal conditions. However, the antifungal activity of ALX decreased as the NaCl concentration increased, whereas antibacterial activity remained unaffected. These findings indicated that electrostatic and hydrophobic balance in ALX is important for antimicrobial activity.

These results evidence that ALX effectiveness is both time- and concentration-dependent, as is CHX [29]. However, ALX kills E. faecalis even at a 100-fold lower concentration than CHX. Also, Silveira et al., whose study aimed to evaluate the antimicrobial activity of ALX alone and combined with N-acetylcysteine (NAC) against two E. faecalis strain biofilms found that ALX showed antimicrobial properties tested at very low concentrations [29]. This outcome might be the result of ALX having a greater affinity for bacterial lipoteichoic acid (LTA), which is a major constituent of the cell wall of gram-positive bacteria, than CHX.

Because of its tissue-dissolving capability as well as its broad antimicrobial action and ability to neutralize toxic products, NaOCl is the most commonly used irrigation solution in endodontic treatment [30]. Although color change and the formation of a dense-brown precipitate, containing PCA, after the reaction of NaOCl and CHX have been reported, Kim et al. reported that the association of ALX/NaOCl did not produce PCA or any precipitate [30]. ALX can be used as an effective canal irrigant sequentially or in combination with NaOCl.

## Conclusions

The results of this study presented that 0.0156% ALX can be a good alternative to CHX as an irrigation solution in endodontic treatment when used for one minute against E. faecalis and *C. albicans*. Laboratory tests are only the first steps in a study of the antimicrobial effects of antiseptics. The bacterial efficiency of irrigation solutions in tube dilution studies may not demonstrate clinical conditions. This study should be followed by a clinical study for a final evaluation of the antimicrobial capability of ALX as a root canal irrigant.
